# Photophysical and DNA‐Binding Properties of Phenoxazine‐Based Push–Pull Type Organic Chromophores: Insights From DFT, Molecular Docking, and Optical Studies

**DOI:** 10.1002/open.202500411

**Published:** 2026-01-05

**Authors:** Praveen Naik, T Aravinda, Kuruvalli Gouthami, Vaddi Damodara Reddy, B Vinay Kumar, Neela H. Yennawar, Kavya S. Keremane

**Affiliations:** ^1^ Department of Chemistry Nitte Meenakshi Institute of Technology, Nitte (Deemed to be University), Bengaluru campus Bengaluru India; ^2^ Department of Biochemistry, Biotechnology REVA University Bengaluru India; ^3^ Department of Chemistry BGS College of Engineering and Technology (BGSCET) Bengaluru India; ^4^ The Huck Institutes of the Life Sciences Pennsylvania State University University Park USA; ^5^ Department of Materials Science and Engineering Pennsylvania State University University Park USA

**Keywords:** biointeractive organic materials, CT‐DNA intercalation, density functional theory (DFT), molecular docking studies, phenoxazine‐based dyads

## Abstract

In this work, we investigate the photophysical and DNA‐binding characteristics of phenoxazine‐based organic dyads comprising donor–acceptor configuration, with the goal of boosting their potential for biointeractive applications. The interaction of these dyes with calf thymus DNA was analyzed using UV spectroscopy, confirming their intercalative binding to DNA base pairs. A straightforward UV spectroscopic approach was developed to elucidate the binding mechanisms between DNA and the dyes. Furthermore, density functional theory (DFT) and time‐dependent DFT calculations provided valuable insights into their electronic structures, optical parameters, and insights of frontier molecular orbitals. Molecular electrostatic potential simulations and mapping helped clarify the electron density distributions that are key for DNA interactions. Finally, molecular docking studies backed up these findings that the structural configuration of nucleic acids significantly influences the binding interactions of push–pull organic dyads, suggesting that these dyes could be promising for use in biological imaging and therapeutic applications.

## Graphical abstract

This study investigates the interaction of phenoxazine‐based organic dyads (**PO1**, **PO2**, **PO4**, and **PO5**) with calf thymus DNA using UV–visible spectroscopy, theoretical HOMO–LUMO analysis, and molecular docking techniques. The results reveal strong DNA‐binding interactions of these dyads, particularly **PO5**, highlighting their potential in biochemical and biophysical applications.

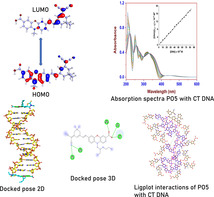

## 1 Introduction

The evolution of supramolecular chemistry has further driven the design of biointeractive materials, making DNA a key target in therapeutic development. Organic compounds play a significant role in modulating their conformation and regulating cellular processes [1, 2, 3]. The organic molecule binds to DNA through intercalation or groove‐binding with *π*‐fused systems, for example, fitting between adjacent DNA base pairs [1, 4]. Lerman et al.'s pioneering studies first characterized the intercalation mechanism in acridine systems, showing that it is driven by noncovalent forces [5]. Numerous compounds, such as metal complexes, Schiff bases, and heterocycles, have since been examined for DNA binding, including transition metal complexes (Pt, Pd, Ru, Cu, and Au), where planar ligands enable moderate to strong DNA‐binding affinity [6, 7, 8, 9, 10, 11]. Transition metal ligands such as acetylacetonato, amine, arene, and Schiff base derivatives, along with polycyclic heteroaromatic compounds, demonstrate substantial DNA‐binding potential through *π*–*π* interactions [12, 13].

Organic dyes are attracting considerable interest across photovoltaics, optoelectronics, and biomedical research due to their unique structural diversity and electronic properties [14, 15, 16, 17, 18]. Organic dyes have proven to be valuable in dye‐sensitized solar cells, where their strong light absorption and efficient electron transfer capabilities are pivotal in boosting device performance [19, 20, 21]. Studies now suggest that the application potential of these dyes extends beyond traditional photovoltaic roles, showing promise in biointeractive domains such as DNA binding and bioimaging, due to their distinctive electronic structures and photophysical characteristics [22, 23, 24, 25].

Organic dye's capability to interact with biomolecules, especially DNA, creates new opportunities for multifunctional applications in biological imaging, diagnostics, and therapeutics [26, 27, 28]. DNA, as the hereditary molecule, is a prime biological target, able to act as a receptor for compounds with potential chemotherapeutic effects due to its roles in genetic information storage and cell replication [29, 30]. DNA interaction studies have shown that organic dyes can bind through intercalative or groove‐binding mechanisms based on structural properties. For instance, *π*‐conjugated systems, such as planar aromatic molecules, can intercalate between DNA base pairs by means of noncovalent forces like *π*–*π* stacking, hydrogen bonding, and hydrophobic interactions [31, 32, 33, 34]. Among these, phenoxazine‐based molecules stand out due to their rigid, tricyclic, and electron‐rich aromatic framework, which enables strong *π*–*π* stacking, hydrogen bonding, and hydrophobic interactions with DNA base pairs. These structural features render phenoxazine scaffolds highly suitable for DNA‐targeted diagnostics and therapeutic development, offering a balance of photostability and tunable electronic properties [35]. While other heterocycles, such as phenothiazines or acridines, have also demonstrated DNA‐binding potential, phenoxazine derivatives often exhibit superior aqueous solubility, reduced photobleaching, and enhanced redox behavior, making them attractive candidates for applications involving biological interfaces. Despite their proven utility in optoelectronics, their biological potential remains underexplored. The results offer new insights into how subtle structural variations in phenoxazine dyes influence DNA binding, setting the stage for future design of targeted sensing and therapeutic agents.

**FIGURE 1:**
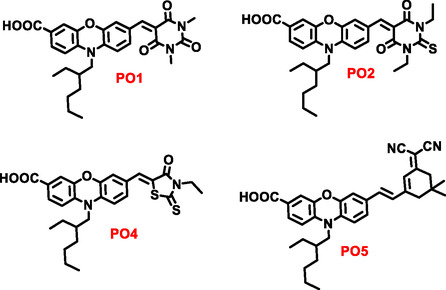
The molecular structures of organic dyads.

This study explores the broader biological potential of our previously reported push–pull‐type phenoxazine‐based organic chromophores (**PO1**, **PO2**, **PO4**, and **PO5**), each featuring different electron‐withdrawing acceptor units [36] (Figure 1) by examining their photophysical behavior and interactions with calf thymus DNA (CT‐DNA). To establish a rational design strategy, careful consideration was given to the selection of electron‐accepting units such as *N*,*N*‐dimethyl barbituric acid, *N*,*N*‐diethyl thiobarbituric acid, 3‐ethylrhodanine, and (3,5,5‐trimethylcyclohex‐2‐enylidene)malononitrile incorporated at the C(3) position of the phenoxazine core [36]. The rationale for selecting these acceptor units is grounded in their distinct electronic properties that modulate intramolecular charge transfer (ICT), conjugation length, and overall dye planarity, all of which influence DNA‐binding affinity and mode. These acceptor groups also impact light absorption, redox behavior, and electron distribution, making them ideal candidates for investigating structure–activity relationships in DNA interactions. These units vary in their electron affinity, heteroatom composition (including O, S, and N‐containing rings), conjugation length, planarity, polarity, and hydrogen bonding potential, which can significantly influence binding affinity toward biological targets, especially in groove binding or electrostatic interactions [36, 37, 38]. This variation allows us to systematically adjust the HOMO–LUMO gap and dipole moment. Their successful exploration in optoelectronic materials, bioimaging probes, and molecular recognition systems further confirms their suitability for studying DNA interactions. By using a diverse range of acceptors, we aimed to see how structural differences in planar *π*‐conjugated systems with polar functional groups affect electronic distribution, optical behavior, stacking interactions with nucleobases, and binding affinity toward CT‐DNA.

To investigate the binding affinity of phenoxazine‐based dyads with CT‐DNA, we employed a combination of experimental and computational approaches. Evaluating the frontier molecular orbitals (HOMO and LUMO) is particularly important because these electronic parameters influence molecular planarity, charge–transfer efficiency, and *π*‐electron distribution, which are the key factors governing intercalative and groove‐binding interactions with DNA. A smaller HOMO–LUMO gap and stronger ICT character typically enhance *π*–*π* stacking ability and electrostatic complementarity with DNA base pairs, thereby providing predictive insight into the dye's binding affinity and mode. Additionally, comparing simulated absorption spectra with experimental UV–Vis data helps establish the reliability of the computational model and clarifies the role of ICT transitions in DNA interaction. Molecular electrostatic potential surface maps further elucidated the spatial distribution of electron density, identifying electropositive and electronegative regions relevant for molecular recognition and DNA interaction. UV–Vis absorption spectroscopy was used to analyze the optical properties of the dyads in chloroform solution, revealing solvatochromic shifts and absorption features pertinent to their structural attributes. Additionally, we utilized AutoDock Vina for molecular docking simulations and PyMOL and COOT for 3D visualization and interaction analysis, highlighting intercalative and groove‐binding modes. Further, BIOVIA Discovery Studio Visualizer for detailed 2D and 3D mapping of ligand–DNA binding. Absorption, distribution, metabolism, excretion, and toxicity (ADMET) and pharmacokinetic properties were assessed using SwissADME and admetSAR, while Molinspiration Cheminformatics was employed for evaluating bioactivity scores [39]. Together, these multifaceted analyses provide a comprehensive understanding of the electronic structure, optical behavior, and biomolecular interactions of the dyads, underscoring their potential as DNA‐interacting chromophores for biological and medicinal applications.

## 2 Results and Discussion

### 2.1 DNA Interaction Studies via UV–Visible Spectroscopy

We conducted DNA interaction studies using UV–visible spectroscopy, focusing on the UV–Vis absorption spectra to gauge how these compounds bind to CT‐DNA in a Tris–HCl buffer (10 mM, pH 7.45). To find the DNA concentration, we measured the absorbance at 260 nm, applying an absorption coefficient of 6600 M^−1^ cm^−1^ [40, 41]. The *A*_260_/*A*_280_ ratio of 1.82 confirmed that our CT‐DNA was of high purity. While keeping the compound concentration steady, we varied the CT‐DNA concentration from 0 to 60 μM during our absorbance titrations. Each run included a 5‐min incubation before we recorded the spectra.

**FIGURE 2:**
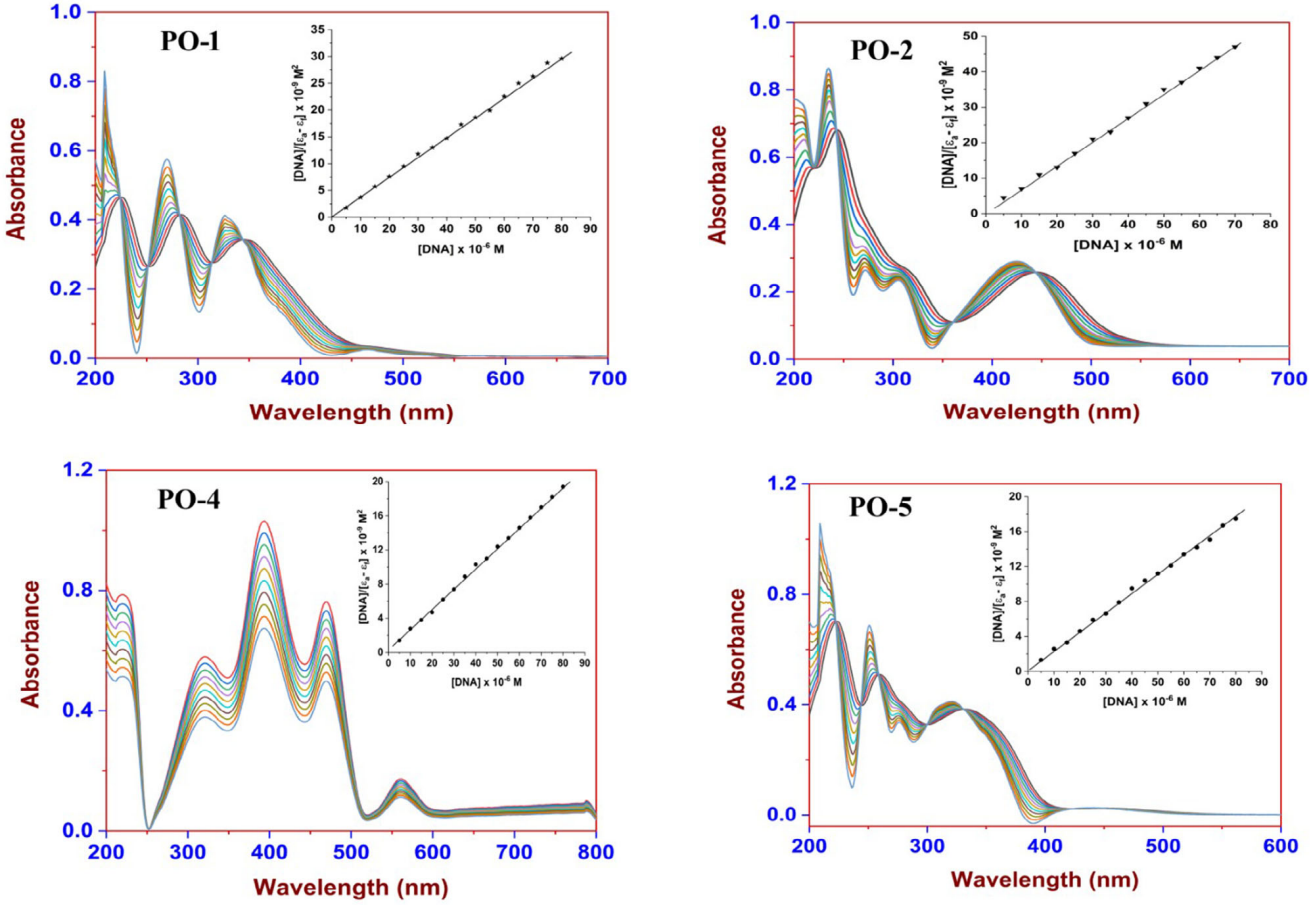
UV–Vis absorption spectra of organic dyes (0.5 mM) in Tris–HCl buffer upon incremental addition of CT‐DNA (0 to 100 μM).

The interactions of compounds **PO1**, **PO2**, **PO4,** and **PO5** with DNA helices were studied using the absorption spectroscopy technique. Intercalative binding of a molecule to DNA is typically indicated by a decrease in absorbance (hypochromism) and a redshift in wavelength, attributed to strong stacking interactions between the molecule's aromatic system and the DNA base pairs. The extent of hypochromia correlates directly with the strength of intercalative interactions, as demonstrated in previous studies [42]. Figure 2 shows the electronic absorption range for the dye molecules with CT‐DNA in Tris buffer at pH of 7.2.

As depicted in Figure 2, the absorption spectra of compounds **PO1**, **PO2**, **PO4**, and **PO5** in combination with CT‐DNA show remarkable hypochromism (H%) and an observable red shift, indicating possible intercalation phenomena [32, 33]. The color coding in the UV–Vis absorption spectra (Figure 2) likely represents incremental concentrations of CT‐DNA added to a fixed concentration of the organic dye solutions (0.5 mM), a standard method to visualize how the absorbance spectrum of a dye changes upon progressive DNA addition, often starting from 0 up to 100 µM DNA. The initial spectrum (light sky blue) corresponds to the dye alone (0 µM DNA). The progressive color shifts show spectra after increasing the DNA concentration. The extent of hypochromia correlates directly with the strength of intercalative interactions, as the **IL (Intraligand)** *π*→π* **transition** is diminished due to the strong stacking interaction between the molecule's aromatic system and the DNA base pairs. This is a common spectroscopic marker for intercalation.

To determine the intrinsic binding constants (**K**_**b**_) of compounds **PO1**, **PO2**, **PO4**, and **PO5** with CT‐DNA, careful absorbance measurements were performed at wavelengths 330, 412, 487, and 326 nm. The resulting intrinsic binding constants (**K**_**b**_) were then calculated for the five compounds, intrinsic binding values of 4.22 × 10^5^ M^−1^ (PO1), 1.16 × 10^4^ M^−1^ (PO2), 4.73 × 10^5^ M^−1^ (PO4), and 2.51 × 10^4^ M^−1^ (PO5), respectively. This establishes a hierarchical order of binding with CT‐DNA as **PO4 > PO1 > PO5 > PO2**. The strength and continuity of the interaction between compounds **PO1**, **PO2**, **PO4**, and **PO5** with CT‐DNA are highly influenced by the planarity of the intercalating molecules. This conclusion implies that the different values of **K**_**b**_ among the four compounds are largely determined by the coordination complexities of the primary compound [30, 43, 44, 45].

### 2.2 Computational Analysis

**FIGURE 3:**
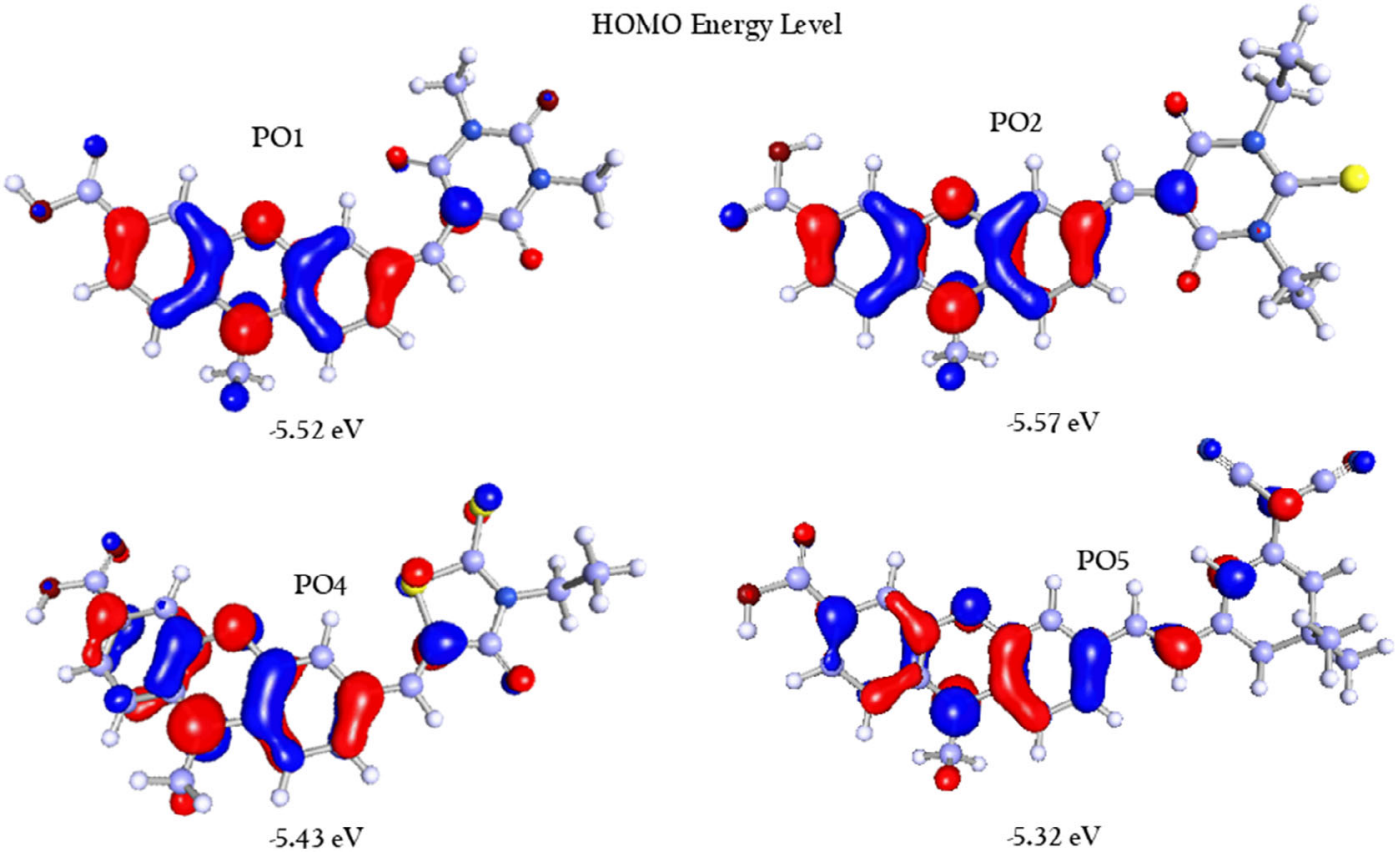
HOMO energy levels of **PO1**, **PO2**, **PO4**, and **PO5** dyes.

**FIGURE 4:**
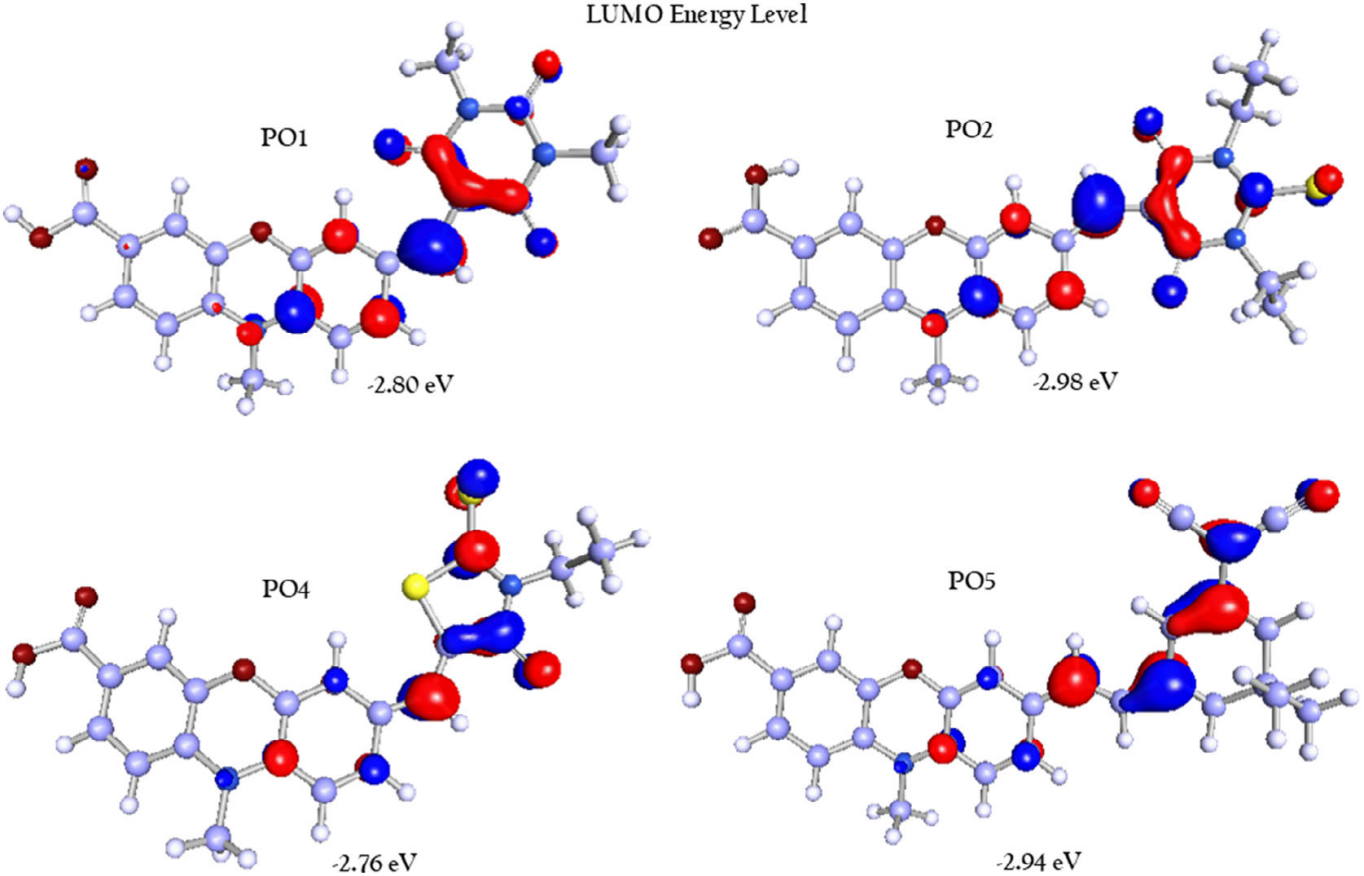
LUMO energy levels of **PO1**, **PO2**, **PO4**, and **PO5** dyes.

To investigate the electronic properties of the organic dyes **PO1**, **PO2**, **PO4**, and **PO5**, density functional theory (DFT) calculations were performed using Biovia Turbomole 2022 [46, 47, 48]. These geometries were further optimized by applying C1 point group symmetry generated by the software, with the B3LYP method and def‐TZVP basis set for all quantum chemical calculations [49, 50, 51, 52]. B3LYP hybrid functional remains widely used and well‐validated for predicting low‐lying singlet excited states in medium‐sized organic molecules, including push–pull dyes such as those studied here. Numerous studies have demonstrated that B3LYP performs reliably in describing optical absorption characteristics and the overall frontier orbital framework in similar donor–acceptor (D–A) systems. The corresponding electron density distributions of the frontier molecular orbitals (FMOs) of the dyads are presented in Figures 3 and 4. It is evident that the electron‐donating nature of the carbazole unit plays a significant role in determining the energy levels of HOMO and spatial charge distribution, thereby affecting interfacial charge transport. Further, FMOs are often utilized in establishing charge transport behavior owing to highly delocalized FMOs facilitating charge mobility through enhancing electronic coupling between neighboring molecules and lowering nuclear reorganization energy. These calculations provided us with a clear picture of how electron density is distributed within the HOMO and LUMO levels, along with optimized geometries in the gas phase. As illustrated in Figures 3 and 4, a distinct charge separation is observed in the frontier molecular orbitals, with the electron density predominantly localized on the phenoxazine donor skeleton, which clearly indicates the electron‐donating ability in the HOMO and shifting toward the acceptor unit in the LUMO.

For **PO1**, the HOMO energy sits at −5.52 eV, while the LUMO energy is at −2.80 eV, resulting in a HOMO–LUMO gap of 2.72 eV. Dyes **PO2**, **PO4**, and **PO5** show similar HOMO–LUMO gaps of 2.59, 2.67, and 2.38 eV, respectively. The movement of electron density toward the acceptor in the LUMO indicates that all these dyes exhibit efficient charge transfer behavior. The calculated HOMO–LUMO separations align closely with experimental electronic data, supporting the potential of **PO1**, **PO2**, **PO4**, and **PO5** for strong interactions with biomolecular targets, such as DNA. The HOMO–LUMO energy gaps of the dyes range from 2.38 eV (**PO5**) to 2.72 eV (PO1), reflecting the degree of ICT between the phenoxazine donor core and various acceptor groups. The HOMO is delocalized over the phenoxazine core, while the LUMO is primarily located on the acceptor moieties, indicating effective donor–acceptor separation, which enhances binding through dipole‐induced interactions with DNA. The relatively smaller gap in **PO5** suggests stronger ICT and higher electron delocalization, facilitating enhanced *π*–*π* stacking and electrostatic interactions with DNA base pairs. This trend aligns with the electron‐withdrawing strength of the dicyano acceptor units in **PO5** induces a deeper LUMO and narrower gap, favoring stronger interactions with electron‐rich nucleobases. Conversely, PO1 and PO4 exhibit wider bandgaps and comparatively weaker ICT, implying a reduced binding affinity. These electronic properties highlight their suitability for molecular recognition and charge transfer applications, as evidenced by the DFT analysis (Table 1).

**TABLE 1:** HOMO, LUMO energies, and HOMO–LUMO gap for compounds.

Compound	HOMO (eV)	LUMO (eV)	HOMO–LUMO gap (eV)
**PO1**	−5.52	−2.80	2.72
**PO2**	−5.57	−2.98	2.59
**PO4**	−5.43	−2.76	2.67
**PO5**	−5.32	−2.94	2.38

**FIGURE 5:**
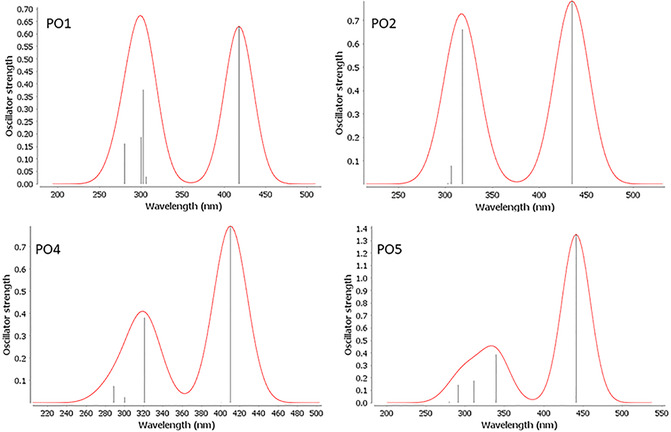
Simulated absorption spectra of dyes in gas phase.

Furthermore, time‐dependent DFT (TD‐DFT) calculations were carried out to gain deeper insights into the vibrational and optical properties of the dyes. The UV–visible absorption spectra of **PO1**, **PO2**, **PO4**, and **PO5** were simulated using the CAM‐B3LYP functional combined with the def‐TZVP basis set [53], as depicted in Figure 5. Based on the adiabatic approximation, TD‐DFT assumes that time nonlocality of the exchange‐correlation (xc) functional may be ignored, i.e., the xc potential at any point in time is a function only of the current electron density. This allows us to use xc functionals parameterized for ground‐state DFT, e.g., the Becke–Perdew and hybrid B3LYP functionals [54, 55]. The choice of the basis set in TD‐DFT calculations continues to be the most significant issue in determining the level of accuracy of results. The choice of the basis set in TD‐DFT calculations continues to be the most significant issue in determining the level of accuracy of results. Notably, TD‐DFT provides good predictions for energy levels of long‐range charge–transfer states. As shown in Figure 5, all dyes exhibit two prominent absorption bands corresponding to *π*–π* transitions and ICT processes within the molecules in the visible light region. The redshift of **PO2** and **PO5** *λ*_max_ values relative to **PO1** and **PO4** suggests increased electron donation in the latter molecules. These results confirm that the chosen basis and functional set yield theoretical findings that align well with experimental results. The simulated absorption spectra (Figure 5) show strong transitions in the UV–visible region (λ_max_ ∼ 300–450 nm), with **PO5** exhibiting the highest oscillator strength (up to 1.3), consistent with its stronger ICT nature. When compared to experimental UV–Vis data, a bathochromic (red) shift is observed upon DNA addition, confirming intercalative binding. For example, **PO2** and **PO5** display notable hypochromism and redshifts, supporting *π*–*π* stacking interactions. The shifts in *λ*_max_ between simulated and experimental spectra can be attributed to the solvent and DNA environment in the latter, which are absent in gas‐phase TD‐DFT calculations. However, the overall trends in relative absorption intensity and position across **PO1‐PO5** remain consistent, validating the computed spectra as supportive of experimental findings. The simulated spectra confirm that the applied computational protocol accurately captures the optical behavior of the push–pull systems under investigation.

**FIGURE 6:**
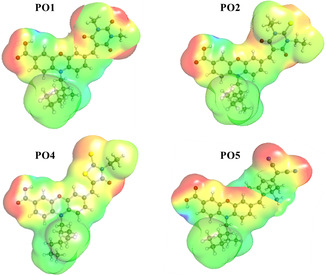
ESP map of **PO1**, **PO2**, **PO4**, and **PO5** dyads.

Figure 6 displays the electrostatic potential (ESP) maps of **PO1**, **PO2**, **PO4**, and **PO5**, illustrating the spatial distribution of charge across the dye molecules. These visualizations highlight how the charge density isosurface corresponds to molecular cavities, revealing key features such as electronegativity differences, dipole orientations, and potential reactive sites. The ESP color gradient follows the order blue > green > yellow > orange > red [56, 57, 58]. In these plots, blue regions indicate areas of high positive potential, generally located around hydrogen atoms or less electronegative moieties, which are prone to electrophilic interactions, while red regions represent zones of high electron density (negative potential), typically associated with electronegative atoms such as oxygen or nitrogen, which may participate in hydrogen bonding or nucleophilic interactions. The intermediate colors (green, yellow, and orange), denoting gradual transitions, represent neutral or moderate potential regions, highlighting the transition between electron‐rich and electron‐poor zones. The contrasting red and blue areas define electron‐rich and electron‐poor zones, establishing local electric fields across the molecular framework. Notably, the ESP maps clearly demonstrate a directional shift of electron density from the donor units toward the acceptor or anchoring groups through spacer segments. This internal charge migration supports effective charge separation, a feature critical for the efficient operation of **PO1**, **PO2**, **PO4**, and **PO5** in optoelectronic applications.

Among all four dyads, **PO2** and **PO5** exhibit more localized and intense regions of negative potential around their acceptor units (notably dicyano and rhodanine‐type moieties), which can facilitate stronger electrostatic and hydrogen bonding interactions with the DNA phosphate backbone or groove regions. In contrast, PO4 shows more evenly distributed ESP, potentially leading to weaker specific electrostatic anchoring but allowing better *π*–*π* stacking intercalation due to its broader conjugation. The distribution and intensity of ESP influence how the chromophore aligns with and binds to DNA through groove‐binding, electrostatic attraction, and intercalative *π*‐stacking. These differences help rationalize the potential of **PO5**, and its localized electronegative regions enhance groove‐specific interactions, possibly along with hydrophobic effects from its bulky substituent. Overall, the insights gained from the ESP analysis provide a deeper understanding of the electronic properties of these push‐pull molecules and promote internal charge separation, supporting efficient donor–acceptor behavior. These electronic features are essential for understanding the binding affinity of these dyads with DNA, as electron‐rich and deficient regions guide molecular recognition, especially in intercalative binding modes. Notably, the spatial arrangement of potentials in **PO5** and **PO2** may explain their relatively higher DNA‐binding constants.

### 2.3 Pharmacokinetic Modeling, Bioactivity Assessment, and Molecular Docking Studies

Phenoxazine scaffolds are well‐known for their redox activity, photophysical properties, and notable biological interactions, including antibacterial and anticancer effects. However, their cytotoxicity is often structure‐dependent and can pose a limitation for therapeutic applications. Similarly, dicyano‐substituted acceptors, commonly used for their strong electron‐withdrawing properties, have also demonstrated variable cytotoxic profiles, particularly in compounds designed for photovoltaic and bioimaging purposes [59]. In this study, molecular docking simulations and absorption, distribution, metabolism, and excretion (ADME) profiling were employed to assess the binding efficiency and pharmacokinetic behavior of phenoxazine‐based push–pull organic chromophores. These molecules were docked with DNA minor groove targets to evaluate their interaction strength, geometry, and potential as DNA‐binding probes.

#### 2.3.1 Pharmacokinetic Properties of Selected Compounds

Key parameters such as the number of rotatable bonds, AlogP values, molecular weight, and the counts of hydrogen bond donors and acceptors play an essential role in determining the pharmacokinetic behavior of a drug. These factors significantly impact the drug's ADME profiles. According to Lipinski's rule of five, compounds with favorable values for these properties are more likely to exhibit good oral bioavailability, highlighting the importance of optimizing molecular weight, lipophilicity (AlogP), flexibility, and hydrogen bonding potential during drug design (Table 2) [60, 61].

**TABLE 2:** Drug likeness properties of selected compounds.

Compound	miLogP	Molecular weight (g/mol)	TPSA (Å^2^)	natoms	nON	nOHNH	nrotb
**PO1**	4.98	505.56	107.46	37	9	1	8
**PO2**	6.08	549.68	122.48	39	8	1	10
**PO4**	6.82	510.67	127.47	35	6	1	9
**PO5**	7.24	543.74	101.81	40	6	5	11

miLogP, Moriguchi LogP (predicted octanol/water partition coefficient); TPSA, topological polar surface area; natoms, total number of atoms in the molecule; nON, number of hydrogen bond acceptors (N and O atoms); nOHNH, number of hydrogen bond donors (OH and NH groups); nrotb, number of rotatable bonds.

#### 2.3.2 Biological Activity

The biological activity scores of the phytochemical compounds, along with reference drugs, were evaluated by Molinspiration Cheminformatics server (calculation of molecular properties and bioactivity score, molinspiration.com). Evaluating biological activity is essential for gaining insights into the active sites and binding mechanisms, which helps in understanding their mode of action. The results of this analysis are summarized in Table 3 [62].

**TABLE 3:** Bioactive properties of synthesized compounds.

Compound	GPCR ligand	Ion channel modulator	Kinase inhibitor	Nuclear receptor ligand	Protease inhibitor	Enzyme inhibitor
**PO1**	−0.16	−0.65	−0.51	0.03	−0.39	−0.04
**PO2**	−0.24	−0.88	−0.60	−0.19	−0.50	−0.16
**PO4**	−0.49	−1.10	−0.59	−0.23	−0.41	−0.08
**PO5**	0.06	−0.35	−0.30	0.37	−0.09	0.22

#### 2.3.3 ADMET/Pharmacokinetic Analysis of Selected Compounds

**FIGURE 7:**
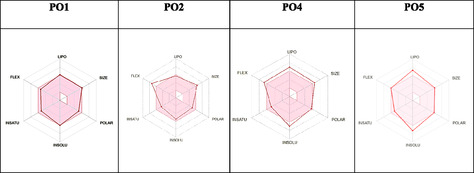
Pharmacological properties of selected compounds.

In drug discovery and development, careful evaluation of ADMET properties is crucial. An effective drug candidate should not only target the intended biological pathway but also display favorable ADMET profiles at therapeutic doses [63]. To support the selection of promising compounds, toxicity prediction and structural similarity analyses were performed using the admetSAR and SwissADME tools, as presented in Figure 7 and Table 4.

**TABLE 4:** Pharmacokinetic properties of synthesized compounds.

Compounds	GI absorption	BBB permeant	P‐gp substrate	CYP1A2 inhibitor	CYP2C19 inhibitor	CYP2C9 inhibitor	CYP2D6 inhibitor	CYP3A4 inhibitor	**Log *K***_**p**_ **(skin permeation)(cm/s)**
**PO1**	High	No	No	No	Yes	Yes	No	Yes	−5.64
**PO2**	Low	No	Yes	No	No	Yes	No	Yes	−4.97
**PO4**	Low	No	Yes	No	No	Yes	No	Yes	−4.24
**PO5**	Low	No	Yes	No	No	No	No	No	−6.57

GI absorption, gastrointestinal absorption; BBB permeant, blood–brain barrier permeation; P‐gp substrate, P‐glycoprotein substrate; CYP1A2 inhibitor, cytochrome P450 1A2 inhibitor; CYP2C19 inhibitor, cytochrome P450 2C19 inhibitor; CYP2C9 inhibitor, cytochrome P450 2C9 inhibitor; CYP2D6 inhibitor, cytochrome P450 2D6 inhibitor; CYP3A4 inhibitor, cytochrome P450 3A4 inhibitor; Log *K*_p_ (skin permeation), skin permeability coefficient (logarithmic form).

Measured as log cm/s.

### 2.4 Molecular Docking Analysis

AutoDock Vina, an advanced open‐source tool for protein‐ligand docking, offers notable improvements in binding mode prediction accuracy and computational efficiency compared to its predecessor. AutoDock Vina 4.2 with an optimized scoring function and support for multithreading, Vina enhances both the speed and reliability of docking simulations [64, 65]. In this study, the molecular docking analysis was performed using AutoDock Vina, and the resulting binding conformations were further visualized and analyzed using PyMOL for three‐dimensional interaction assessment and COOT (Crystallographic Object‐Oriented Toolkit) for detailed inspection and refinement of molecular interactions at the active site. These tools enabled the identification of key hydrogen bonding interactions, *π*–*π* stacking and spatial complementarity between the ligands and the DNA‐binding pocket. The cyan‐colored **PO1**, magenta‐colored **PO2**, green‐colored **PO4**, and blue‐colored **PO5** display an extended conformation along the DNA groove, forming a *π*–*π* stacking interaction with the DNA bases. All four compounds interact with DNA primarily through intercalation, stabilized by *π*–*π* stacking and hydrogen bonding. Receptor and ligand structures were initially prepared by converting PDB files into the PDBQT format. Docking parameters were set with a grid box size of 40 × 40 × 40 Å and a grid spacing of 0.572 Å, defining the search space. The grid center coordinates were specified as *x* = 18.421, *y* = 12.432, and *z* = 13.853. Following grid setup, ligand log files were generated to facilitate docking score calculations using the Cygwin platform. Binding interactions and energies were subsequently analyzed through PyMOL, focusing on key hydrogen bond interactions with active site residues. Further visualization of the ligand–DNA interactions was carried out in both 2D and 3D formats using BIOVIA Discovery Studio Visualizer.

**FIGURE 8:**
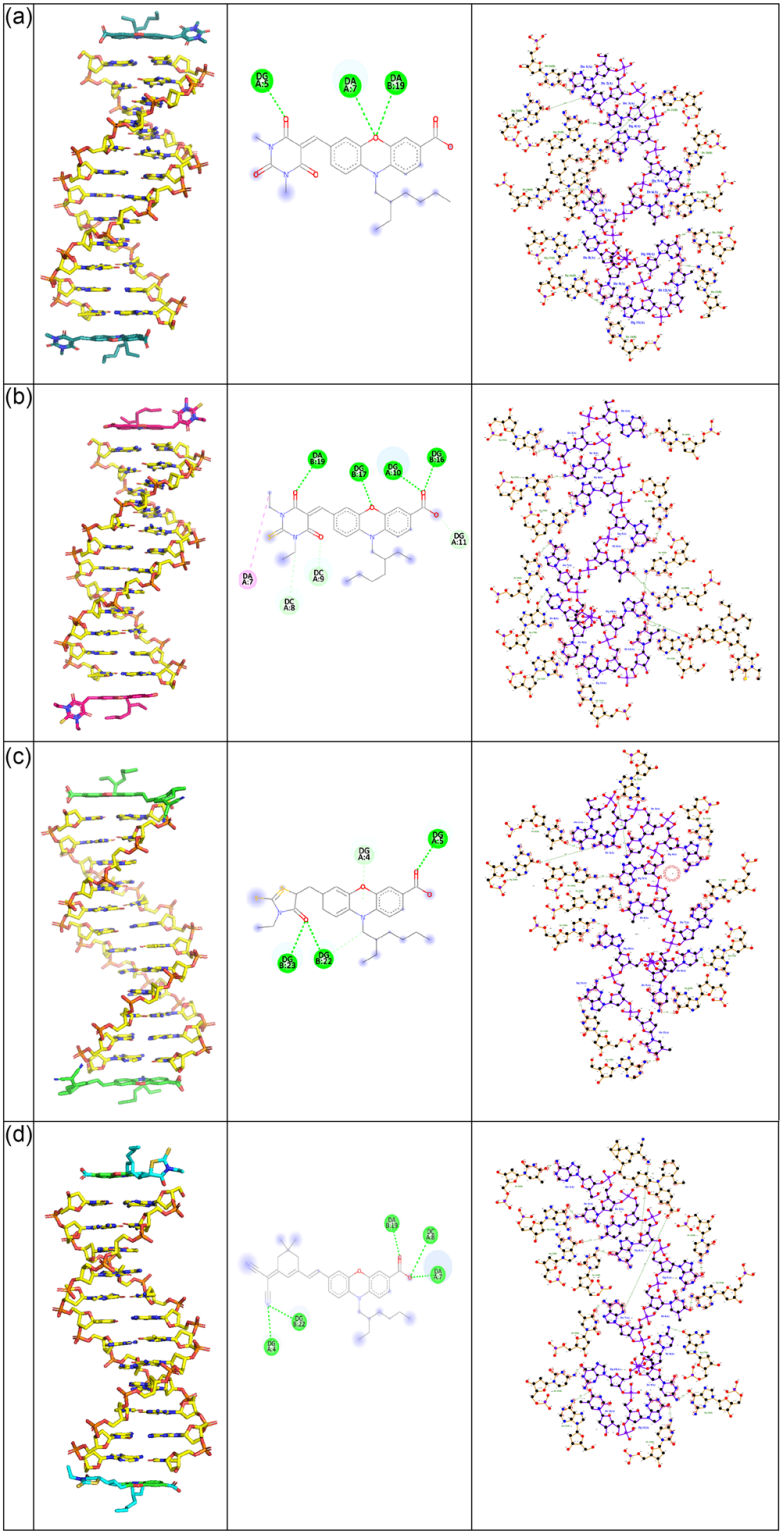
Docked pose 2D, 3D, and Ligplot interactions of compounds (a) **PO1**, (b) **PO2**, (c) **PO4**, and (d) **PO5** with CT–DNA (A‐B) respectively.

Docking studies revealed that the selected compounds exhibited strong binding affinities toward CT‐DNA A–B and their binding parameters are given in Table S1. Five types of interaction potentials typically represent the receptor binding pocket, such as van der Waals interactions for hydrogen atoms and heavy atoms, optimized electrostatics, hydrophobic interactions, and lone‐pair‐based potentials that account for directional hydrogen bonding preferences. These energy terms are derived from the all‐atom vacuum force field ECEPP/3, with additional functions to estimate solvation free energy and entropic contributions. The binding score for each compound was calculated using the following equation:

(1) $$E_{\mathrm{bind}}=E_{\mathrm{int}}+T\text{Δ}S_{\mathrm{Tor}}+E_{\mathrm{VW}}+\alpha 1E_{\mathrm{el}}+\alpha 2E_{\mathrm{hb}}+\alpha 3E_{\mathrm{hp}}\alpha 4E_{\mathrm{sf}}$$

where *E*_vw_ is van der Waals energy, *E*_el_ is electrostatic energy, *E*_hb_ is hydrogen bond energy, *E*_hp_ is hydrophobic interaction energy, *E*_sf_ is solvation free energy (polar and nonpolar atoms), *E*_int_ is internal strain of the organic ligand, Δ*S*_Tor_ is conformational entropy loss on binding, and *α*_1_–*α*_4_ are empirical scaling constants. Each organic dyad was docked into the binding pocket, and a binding score was assigned based on this composite energy evaluation. The organic ligand **PO1** (Figure 8a), showed a docking score of –8.7 kcal/mol, forming three hydrogen bonds with A:DG5, A:DA7, and B:DA19, with bond distances recorded at 1.95, 2.45, and 2.52 Å. The dyad **PO2** (Figure 8b) demonstrated a docking score of −8.1 kcal/mol, forming eight hydrogen bonds with residues B:DA19, A:DG10, B:DG16, B:DG17, A:DG11, A:DC9, A:DA17, and A:DC8. The bond distances ranged between 1.87 and 5.22 Å. The third compound, also labeled **PO4** (Figure 8c), exhibited a docking score of −8.0 kcal/mol, forming four hydrogen bonds with A:DG5, A:DG4, B:DG22, and B:DG23, at bond distances between 2.29 and 2.66 Å. The second lead compound, **PO5** (Figure 8d), achieved a highest docking score of −10.7 kcal/mol, establishing five hydrogen bonds with A:DA7, B:DA19, A:DC8, A:DG4, and B:DG22, with bond lengths spanning from 2.05 to 3.24 Å.

Among the four studied compounds, **PO5** exhibited the strongest binding affinity toward CT‐DNA, with a binding energy of −10.7 kcal/mol, indicating the most stable ligand–DNA complex. Despite having fewer hydrogen bonds than **PO2**, the significantly higher binding affinity of **PO5** suggests that additional noncovalent interactions such as *π*–*π* stacking, van der Waals forces, or hydrophobic interactions may contribute to its superior binding strength. **PO5**, featuring a strong electron‐withdrawing malononitrile unit with two cyano (–CN) groups, significantly enhances the electrophilicity of the acceptor moiety, thereby promoting stronger *π*–*π* stacking and electrostatic interactions with the nucleobases of DNA. Its extended *π*‐conjugated system further facilitates efficient intercalation between DNA base pairs. Additionally, the trimethylcyclohexyl group imparts hydrophobicity and steric bulk, contributing to enhanced van der Waals and hydrophobic interactions within the DNA grooves. These structural attributes are consistent with the observed highest binding affinity of **PO5**, confirming its superior potential for DNA binding through a combination of electronic effects, *π*‐conjugation, and steric compatibility.

## 3 Conclusion

This study examined the DNA‐binding behavior of D–A phenoxazine‐based chromophores through combined spectroscopic and computational approaches. UV–Vis absorption titration confirmed intercalative binding with CT‐DNA and enabled the determination of binding constants. Computational calculations provided insight into the electronic structures, particularly HOMO–LUMO distributions, while ESP mapping highlighted electron‐rich and electron‐deficient regions relevant to DNA recognition. Molecular docking studies revealed strong binding energies for all four dyads, with **PO5** exhibiting the highest affinity (−10.7 kcal/mol), attributed to its strong electron‐withdrawing malononitrile acceptor and extended *π*‐conjugation. Pharmacokinetic modeling and in silico bioactivity profiling of **PO5** reveal a favorable interaction landscape, consistent with its strong binding affinity and stability in DNA complexes observed through spectroscopic and computational studies. The integrated evidence from UV–Vis titration, molecular docking, frontier molecular orbital analysis, and ESP mapping suggests that **PO5** possesses an optimal electronic structure and molecular topology conducive to DNA intercalation. While these findings position **PO5** as a promising lead candidate for DNA‐targeted therapeutic development, its potential cytotoxicity and biological efficacy must be rigorously validated through subsequent in vitro and in vivo studies.

## Supporting Information

Additional supporting information can be found online in the Supporting Information section. **Supporting Table S1:** The binding affinity of CT‐DNA against compounds.

## Conflicts of Interest

The authors declare no conflicts of interest.

## Supporting information

Supplementary Material

## Data Availability

The data that support the findings of this study are available in the Supporting Information of this article.
