# Supplementary material for: Photophysical and DNA‐Binding Properties of Phenoxazine‐Based Push–Pull Type Organic Chromophores: Insights From DFT, Molecular Docking, and Optical Studies
Source: ChemistryOpen. 2026 Jan 5;15(1):e202500411. doi: 10.1002/open.202500411 (PMC12771586; doi:10.1002/open.202500411)
Supplement: Supplementary file 1 — Supplementary Material [file OPEN-15-e202500411-s001.pdf]

Electronic supplementary information

Photophysical and DNA Binding Properties of Phenoxazine-based Push-Pull Type Organic Chromophores: Insights From DFT, Molecular Docking, and Optical Studies

Praveen Naik [1, *], Aravinda T [1], Kuruvalli Gouthami [2], Vaddi Damodara Reddy [2], Vinay Kumar B [3], Neela H. Yennawar [4], Kavya S. Keremane [5, *]

[1]*Department of Chemistry, Nitte Meenakshi Institute of Technology, Yelahanka, Bengaluru- 560064.*

[2]*Department of Biotechnology, REVA University, Bengaluru-560064, Karnataka, India.*

[3]*Department of Chemistry, BGS College of Engineering and Technology, Mahalakshmipuram, Bengaluru – 560086*

[4] *The Huck Institutes of the Life Sciences, Pennsylvania State University, University Park, Pennsylvania 16802, United States.*

[5]*Department of Materials Science and Engineering, The Pennsylvania State University, University Park, PA, 16802, USA.*

Corresponding author's email: praveennaik018@gmail.com, kxk5889@psu.edu

Experimental Section

1. Materials and methods

The starting materials such as 10*H*-phenoxazine, 2-ethylhexyl bromide, *N*,*N*-dimethyl barbituric acid, *N*,*N*-diethyl thiobarbituric acid, malononitrile, 3-ethylrhodanine, (3,5,5-trimethylcyclohex-2-enylidene)malononitrile and *2*-oxindole were procured from Sigma-Aldrich and Alfa Aesar companies. All the solvents used in the reactions were of synthetic grade (Merck, Loba Chemie, and Spectrochem companies) and they were purified by further drying and distillation process. All the reactions were carried under an inert (argon) atmosphere and the reaction completion was monitored by the TLC technique. The designed dyes were synthesized by using standard synthesis protocol. The target dyes and their intermediates were purified using recrystallization or column chromatographic separation techniques.

2. Synthetic methods

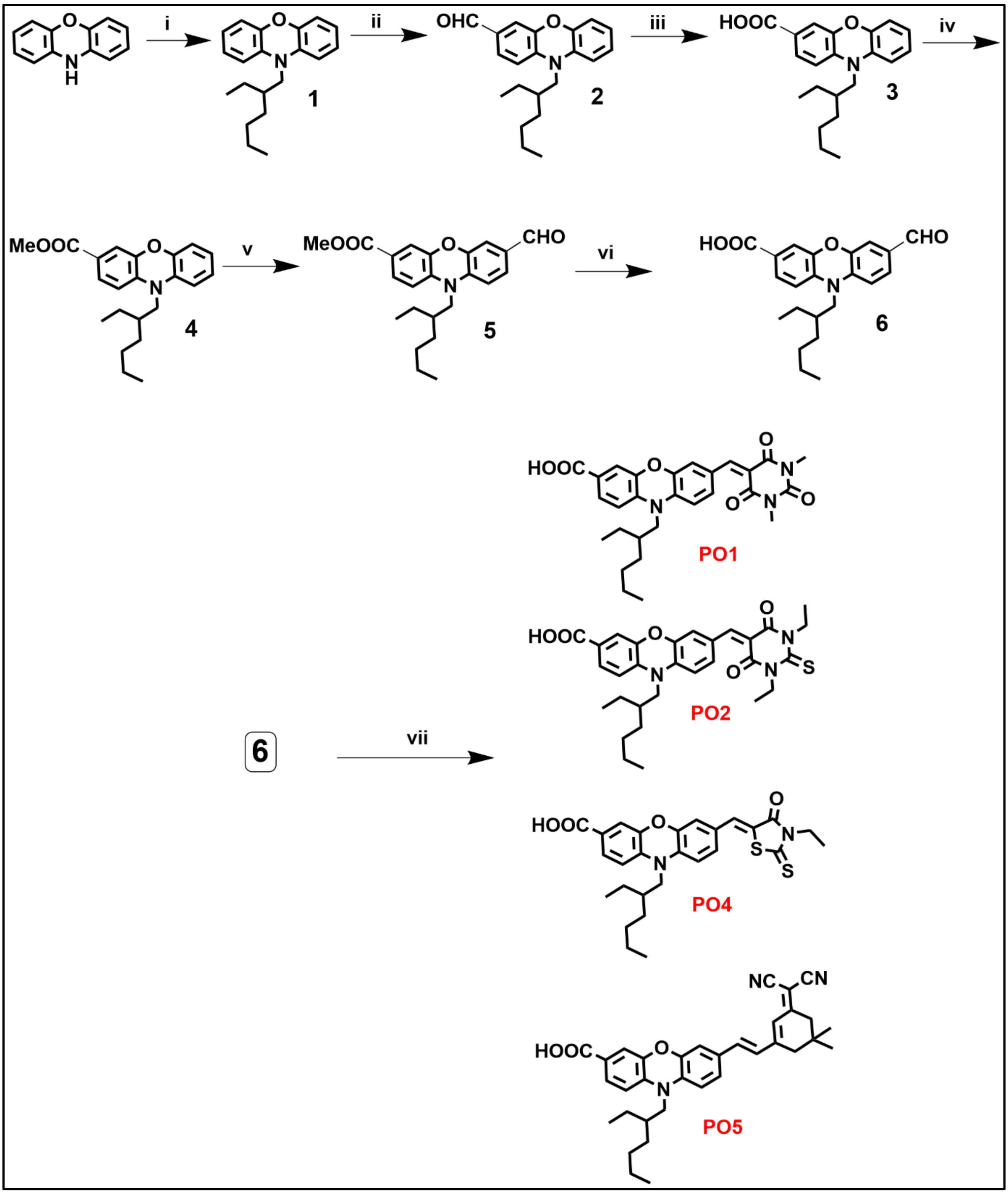

*Synthesis of 10-(2-ethylhexyl)-10H-phenoxazine (**1**)*

A mixture of phenoxazine (0.5 g, 2.72 mmol), NaH (1.9 g, 8.16 mmol) was dissolved in a minimum amount of DMF (8 mL) and stirred at room temperature for half an hour under an inert atmosphere. Later, 2-ethylhexyl bromide (0.58 mL, 3.27 mmol) was added into the reaction mixture and continued stirring at room temperature for 12 h. The reaction progress was monitored using TLC. After completion of the reaction, the reaction mixture was cooled and poured into crushed ice (100 mL) and neutralization was done using a saturated solution of ammonium chloride. The residue formed was extracted with ethyl acetate (50 mL x 4) and the organic layer was dried over sodium sulphate and evaporated under reduced pressure. The impure residue was later purified by column chromatography on silica gel (100-200 mesh and hexane: EtOAc eluent) to yield a colorless liquid as a product. Yield: 97%.

^{1}H NMR (400 MHz, $CDCl_3$, δ ppm): 6.77-6.73 (m, 2H), 6.61-6.60 (d, 4H), 6.51-6.49 (m, 2H), 3.39-3.37 (d, 2H), 1.88-1.85 (m, 1H), 1.36-1.29 (m, 8H), 0.90-0.87 (t, 6H). Anal. Calcd. for $C_{20}H_{25}NO$: C, 81.31; H, 8.53; N, 4.74; and found C, 80.89; H, 8.29; N, 4.06.

*Synthesis of 10-(2-ethylhexyl)-10H-phenoxazine-3-carbaldehyde (**2**)*

In a cleaned RB flask, freshly distilled DMF (0.13 mL, 1.69 mmol) was taken and cooled at –3 to 4 ^{o}C. Then, phosphorous oxychloride, $POCl_3$ (0.09 mL, 1.01 mmol) was added drop-wise with constant stirring at the same temperature under argon atmosphere to obtain a glassy white salt. To this mixture, 10-(2-ethylhexyl)-10*H*-phenoxazine (**1,** 0.1 g, 0.33 mmol) dissolved in dichloroethane (2 mL) was added. The reaction mixture was refluxed at 95 ºC for 12 h. After completion of the reaction, the reaction mass was cooled to room temperature and poured into 100 mL crushed ice, and subsequently basified by using a 5 M NaOH solution. The product formed was extracted with ethyl acetate (50 mL x 3) and the organic layer was dried over sodium sulphate and evaporated under reduced pressure. The impure residue was later purified by column chromatography on silica gel (100-200 mesh) to yield a light brown solid (**2**). Yield: 79 %. Melting point: 112-114 °C.

^{1}H NMR (400 MHz, $CDCl_3$, δ ppm): 9.68 (s, 1H), 7.32-7.30 (m, 1H), 7.10-7.09 (d, 1H), 6.83-6.80 (m, 1H), 6.75-6.74 (d, 1H), 6.68-6.66 (d, 1H), 6.61-6.58 (t, 1H), 3.50-3.48 (d, 2H), 1.92-1.89 (m, 1H), 1.50-1.31 (m, 8H), 0.97-0.94 (t, 3H), 0.92-0.89 (t, 3H). **^{13}C NMR** (400 MHz, $CDCl_3$, δ ppm): 189.63, 145.18, 144.77, 140.16, 132.09, 129.80, 128.43, 123.70, 122.55, 115.84, 114.41, 112.82, 111.23, 47.77, 36.64, 30.68, 28.75, 24.10, 23.07, 14.03,

10.97. Anal. Calcd. for $C_{21}H_{25}NO_2$: C, 77.98; H, 7.79; N, 4.33; and found C, 77.90; H, 7.71; N, 4.23.

*Synthesis of 10-(2-ethylhexyl)-10H-phenoxazine-3-carboxylic acid (**3**)*

In a cleaned RB flask, sodium hydroxide (0.67 g, 17 mmol) was taken and slowly dissolved in 100 mL of ethanol by stirring under an ice bath. Further, the silver oxide (0.35 g, 1.54 mmol) was suspended in this solution and to this mixture 10-(2-ethylhexyl)-10*H*-phenoxazine-3-carbaldehyde (**2**, 0.1 g, O.309 mmol) dissolved in 10 mL of toluene solution was slowly added with constant stirring at RT. The stirring was continued for 16-18 h under an argon atmosphere. After completion of the reaction, the reaction mixture was filtered through a celite bed. Further, the solvent of the filtrate was removed under vacuum and the obtained residue was washed with distilled water. The product was extracted with ethyl acetate (4 × 100 mL) and the organic phase was washed with 10% sodium bicarbonate solution. Pre-cooled 10 % HCl was added to the collected aqueous layer and the pH of the solution was brought to slightly acidic. The precipitated solid was filtered and dried. The residue was purified using column chromatography on 200-400 mess silica using 2:1 hexane/ethyl acetate mixture as the mobile phase to get a pale-yellow solid. Yield: 77 %. Melting point: 138-140 ºC.

^{1}H NMR (400 MHz, $CDCl_3$, δ ppm): 9.72 (s, 1H), 7.61-7.59 (m, 1H), 7.36-7.28 (m, 3H), 7.13-7.12 (d, 1H), 6.67-6.61 (m, 2H), 3.55-3.54 (d, 2H), 1.91-1.89 (m, 1H), 1.51-1.33 (m, 8H), 0.99-0.95 (t, 3H), 0.93-0.89 (t, 3H). **^{13}C NMR** (400 MHz, $CDCl_3$, δ ppm): 189.70, 170.09, 145.17, 144.30, 138.59, 137.35, 130.93, 128.11, 127.08, 123.00, 117.02, 114.90, 112.14, 47.99, 36.64, 30.65, 28.72, 24.09, 23.03, 14.01, 10.95. Anal. Calcd. for $C_{21}H_{25}NO_3$: C, 74.31; H, 7.42; N, 4.13; and found C, 74.29; H, 7.41; N, 4.03.

*Synthesis of methyl 10-(2-ethylhexyl)-10H-phenoxazine-3-carboxylate (**4**)*

In a dry RB flask, 10-(2-ethylhexyl)-10*H*-phenoxazine-3-carboxylic acid (**3**, 1 g, 3.39 mmol) was dissolved in 15 mL of dry methanol and 2 drops of conc. H_2SO_4 was added. The reaction mixture was further refluxed for 3 h. After completion of the reaction, the product was cooled to RT and the solvent methanol was removed under vacuum using a rota evaporator. The residue was extracted with ethyl acetate (3 × 30 mL) and the organic layer was washed with a 10 % sodium carbonate solution, followed by water. Then, it was dried

using sodium sulfate and the solvent was removed under vacuum to get a pale-yellow liquid. Yield: 90 %.

^{1}H NMR (400 MHz, $CDCl_3$, δ ppm): 7.51-7.49 (m, 1H), 7.24-7.24 (d, 1H), 6.82-6.78 (m, 1H), 6.71-6.69 (m, 1H), 6.65-6.64 (d, 1H), 6.57-6.55 (d, 1H), 6.51-6.49 (d, 1H), 3.86 (s, 3H), 3.45-3.43 (d, 2H), 1.90-1.87 (m, 1H), 1.33-1.32 (m, 8H), 0.96-0.91 (t, 6H). **^{13}C NMR** (400 MHz, $CDCl_3$, δ ppm): 166.35, 144.93, 144.43, 138.58, 132.72, 126.19, 123.58, 122.09, 121.94, 116.13, 115.66, 112.51, 111.08, 51.79, 47.63, 36.60, 30.70, 29.73, 28.76, 24.11, 23.08, 14.15, 14.04, 10.97. Anal. Calcd. for $C_{22}H_{27}NO_3$: C, 74.76; H, 7.70; N, 3.96; and found C, 74.25; H, 7.67; N, 3.88.

*Synthesis of methyl 10-(2-ethylhexyl)-7-formyl-10H-phenoxazine-3-carboxylate (**5**)*

In a cleaned dry RB flask, freshly distilled DMF (4.8 mL, 62.2 mmol), $POCl_3$ (5.8 mL, 62.2 mmol) was added slowly with constant stirring maintained at 0 ºC under an argon atmosphere. Then, a solution of methyl 10-(2-ethylhexyl)-10*H*-phenoxazine-3-carboxylate (**4**, 0.5 g, 1.41 mmol) dissolved in 5 mL of dichloroethane was added to the above reaction mixture with constant stirring. The reaction flask was allowed to attain room temperature and then heated at 90 ºC for 12 h. The reaction mass was quenched in ice-cold water. Its pH was adjusted to 1.0 by adding a 5 M sodium hydroxide solution. The solution was extracted with ethyl acetate (4 x 50 mL) and dried over anhydrous sodium sulfate. The solvent was removed under a vacuum to get a pale-orange liquid. Yield: 93 %.

^{1}H NMR (400 MHz, $CDCl_3$, δ ppm): 9.71(s, 1H), 7.54-7.52 (m, 1H), 7.34-7.28 (m, 2H), 7.12-7.11 (m, 1H), 6.65-6.59 (m, 2H), 2.94 (s, 1H), 3.53-3.51 (d, 2H), 1.91-1.87 (m, 1H), 1.42-1.30 (m, 8H), 0.97-0.94 (t, 3H), 0.92-0.88 (t, 3H). **^{13}C NMR** (400 MHz, $CDCl_3$, δ ppm): 189.63, 166.00, 145.15, 144.26, 138.77, 136.56, 130.78, 128.16, 126.22, 124.01, 116.57, 114.75, 112.10, 111.96, 51.99, 47.93, 36.63, 30.65, 28.72, 24.08, 23.02, 14.00, 10.94.

*Synthesis of 10-(2-ethylhexyl)-7-formyl-10H-phenoxazine-3-carboxylic acid (**6**)*

In a cleaned dry RB flask, the intermediate (**5**) was taken and dissolved in 10 mL of methanol. To this solution, 0.25 g of $LiOH.H_2O$ dissolved in 10 mL of water was added and stirred at 80 ^oC for 8 h. Then, the methanol was removed and the solution was diluted with 100 mL of water. Further, the pH of the solution was made to just be acidic by adding pre-cooled 10 % HCl. The precipitated solid was filtered off and purified using column

chromatography technique on 200-400 mess silica using 2:1 hexane/ethyl acetate mixture. Brown color solid, yield 62%. Melting point: 198-200 ºC.

^{1}H NMR (400 MHz, $CDCl_3$, δ ppm): 12.69 (s, 1H), 9.68 (s, 1H), 7.46-7.42 (m, 2H), 7.10-7.08 (d, 2H), 7.06-7.04 (d, 1H), 6.98-6.90 (m, 1H), 3.66-3.64 (d, 2H), 1.80-1.79 (m, 1H), 1.42-1.20 (m, 8H), 0.89-0.80 (t, 6H). **^{13}C NMR** (400 MHz, $CDCl_3$, δ ppm): 190.54, 166.83, 166.67, 166.60, 144.77, 144.41, 144.11, 144.02, 138.78, 138.44, 137.12, 136.51, 130.78, 128.85, 126.70, 124.82, 124.28, 116.06, 115.79, 114.28, 113.59, 113.46, 113.19, 36.27, 30.24, 30.14, 28.42, 23.70, 22.96, 14.24, 11.16. Anal. Calcd. for $C_{22}H_{25}NO_4$: C, 71.91; H, 6.86; N, 3.81; and found C, 71.90; H, 6.82; N, 3.79.

General synthetic procedure for dyes ***PO_{1-2}***

The starting material 10-(2-ethylhexyl)-7-formyl-10*H*-phenoxazine-3-carboxylic acid (**6**, 0.2 g, 0.54 mmol) was dissolved in 10 mL of absolute methanol and stirred at room temperature for 0.5 h. Then, respective clear solution of *N,N*-dimethyl barbituric acid (0.1 g, 0.65 mmol, 1.2 eq) or *N,N*-diethyl thiobarbituric acid (0.13 g, 0.65 mmol, 1.2 eq) dissolved in 5 mL of methanol was added to it slowly with constant stirring. Further, the reaction mass was stirred at 65 ^oC for 10 h. The precipitated solid was filtered and washed with pre-cooled methanol. The obtained dyes were purified by column chromatography with a 2:1 hexane:ethyl acetate solvent system.

*7-((1,3-Dimethyl-2,4,6-trioxotetrahydropyrimidin-5(2H)-ylidene)methyl)-10-(2-ethylhexyl)-10H-phenoxazine-3-carboxylic acid (**PO_1**)*

Pinkish red solid, Yield 87 %. Melting point: 228-230 ºC. **^{1}H NMR** (400 MHz, DMSO-d6, δ ppm): 12.74 (s, 1H), 8.10-8.08 (d, 2H), 7.71-7.69 (d, 1H), 7.46-7.44 (d, 1H), 7.12 (s, 1H), 6.94-6.89 (m, 2H), 3.68-3.67 (d, 2H), 3.22 (s, 6H), 1.36-1.23 (m, 9H), 0.89-0.81 (t, 6H). **^{13}C NMR** (400 MHz, DMSO-d6, δ ppm): 166.54, 162.99, 161.30, 154.87, 151.41, 144.23, 143.23, 138.42, 136.22, 135.79, 126.45, 126.40, 125.22, 119.82, 116.00, 114.42, 113.79, 112.80, 47.32, 36.49, 31.15, 30.16, 29.07, 28.45, 23.73. Anal. Calcd. for $C_{28}H_{31}N_3O_6$: C, 66.52; H, 6.18; N, 8.31 and found C, 66.50; H, 6.11; N, 8.26. **FT-IR (ATR), υ** cm^{-1}: 2961 (C-H stretch), 1670 (C=O stretch), 1558, 1494 (C=C). **Mass (m/z):** Calculated: 505.56; Obtained (M-H): 504.25.

*7-((1,3-Diethyl-4,6-dioxo-2-thioxotetrahydropyrimidin-5(2H)-ylidene)methyl)-10-(2-ethylhexyl)-10H-phenoxazine-3-carboxylic acid (**PO_2**)*

Purple solid, Yield 87%. Melting point: 228-230 °C. ^{1}**H NMR** (400 MHz, DMSO-d6, δ ppm): 12.72 (s, 1H), 8.17-8.13 (m, 2H), 7.80-7.78 (d, 1H), 7.48-7.45 (m, 1H), 7.16 (s, 1H), 6.99-6.95 (m, 2H), 4.45-4.41 (q, 4H), 3.73-3.71 (d, 2H), 1.37-1.34 (m, 1H), 1.24-1.20 (m, 6H), 1.19-0.91 (m, 8H), 0.89-0.81 (t, 6H). ^{13}**C NMR** (400 MHz, DMSO-d6, δ ppm): 178.89, 166.50, 161.27, 159.04, 156.95, 144.29, 144.02, 143.26, 139.36, 137.32, 136.52, 135.41, 126.70, 126.45, 125.62, 119.94, 116.09, 114.35, 114.08, 113.48, 112.96, 47.42, 43.91, 43.41, 36.55, 30.14, 28.44, 23.71, 22.98, 14.27, 12.68, 12.63, 11.19. Anal. Calcd. for $C_{30}H_{35}N_3O_5S$: C, 65.55; H, 6.42; N, 7.64 and found C, 65.51; H, 6.39; N, 7.26. **FT-IR (ATR), υ** cm^{-1}: 2961 (C-H stretch), 1685 (C=O stretch), 1540, 1490 (C=C). **Mass (m/z):** Calculated: 549.68; Obtained (M+H): 550.25.

*Synthesis of (Z)-7-((3-ethyl-4-oxo-2-thioxothiazolidin-5-ylidene)methyl)-10-(2-ethylhexyl)-10H-phenoxazine-3-carboxylic acid (**PO$_4$**)*

A mixture of 10-(2-ethylhexyl)-7-formyl-10*H*-phenoxazine-3-carboxylic acid (**6**, 0.3 g, 0.81 mmol) 3-ethylrhodanine (0.15 g, 0.97 mmol), and NH_4OAc (0.69 g, 8.98 mmol) was taken in a dry RB flask and dissolved in 10 mL of glacial acetic acid and further stirred at 110 ^{o}C for 12 h under an argon atmosphere. The reaction completion was monitored by the TLC method. After completion of the reaction, the content was cooled to room temperature and then it was poured into 100 g of crushed ice. The obtained red solid was filtered and dried. The product was purified by column chromatography using silica gel (200-400 mesh) and $CHCl_3$:CH_3OH (10:1) as the mobile phase to get a red color fine powder as product, yield 84 %, melting point: 230-232 ^{o}C.

^{1}**H NMR** (400 MHz, DMSO-d6, δ ppm): 12.57 (s, 1H), 7.55 (s, 1H), 7.44-7.42 (m, 1H), 7.11-7.04 (m, 2H), 6.89-6.71 (m, 3H), 4.05-4.03 (d, 2H), 3.59-3.33 (m, 2H), 1.31 (m, 1H), 1.22-0.16 (m, 11H), 0.89-0.81 (t, 6H). ^{13}**C NMR** (400 MHz, DMSO-d6, δ ppm): 192.75, 192.40, 167.09, 166.68, 166.60, 158.78, 144.65, 144.31, 144.14, 143.91, 140.47, 137.14, 136.41, 135.73, 135.50, 132.51, 126.88, 126.70, 126.57, 124.69, 124.35, 119.84, 119.25, 117.26, 116.77, 116.01, 114.13, 113.22, 47.30, 35.44, 30.20, 28.45, 23.75, 22.98, 14.26, 12.38, 11.20. Anal. Calcd. for $C_{27}H_{30}N_2O_4S_2$: C, 63.50; H, 5.92; N, 5.49 and found C, 63.48; H, 5.91; N, 5.48. **FT-IR (ATR), υ** cm^{-1}: 2960 (C-H stretch), 1682 (C=O stretch), 1582, 1501 (C=C). **Mass (m/z):** Calculated: 510.67; Obtained (M-H): 509.20

*Synthesis of (E)-7-(2-(3-(dicyanomethylene)-5,5-dimethylcyclohex-1-en-1-yl)vinyl)-10-(2-ethylhexyl)-10H-phenoxazine-3-carboxylic acid (**PO$_5$**)*

A mixture of 10-(2-ethylhexyl)-7-formyl-10*H*-phenoxazine-3-carboxylic acid (**6**, 0.2 g, 0.54 mmol), (3,5,5-trimethylcyclohex-2-enylidene) malononitrile (0.4 g, 2.17 mmol), and piperidine (0.5 mL) was taken in a dry RB flask and dissolved in 10 mL of acetonitrile and further stirred at 80-85 ^{o}C for 12 h under an argon atmosphere. The reaction completion was monitored by the TLC method. After completion of the reaction, the content was cooled to room temperature, and then it was poured into 100 g of crushed ice. Further, the product was extracted with ethyl acetate (3 × 30 mL). The crude product was purified by column chromatography using silica gel (200-400 mesh) and $CHCl_3$:CH_3OH (10:1) as the mobile phase to get pink color fine powder as product, yield 81 %, melting point: 280-282 ^{o}C.

^{1}H NMR (400 MHz, DMSO-d6, δ ppm): 12.74 (s, 1H), 7.40-7.38 (m, 1H), 7.28 (s, 1H), 7.24 (s, 1H), 7.13 (s, 2H), 7.08 (s, 1H), 6.83 (s, 1H), 6.80-6.76 (m, 2H), 4.90-3.88 (d, 2H), 1.52 (s, 4H), 1.40-1.16 (m, 8H), 1.01 (s, 6H), 0.89-0.80 (t, 6H). **^{13}C NMR** (400 MHz, DMSO-d6, δ ppm): 164.47, 162.88, 162.74, 156.69, 150.76, 145.08, 144.47, 144.44, 134.76, 134.21, 125.85, 129.53, 128.71, 127.81, 125.20, 125.05, 124.20, 123.04, 122.95, 122.77, 122.58, 122.51, 120.71, 115.80, 111.34, 110.60, 110.53. Anal. Calcd. for $C_{34}H_{37}N_3O_3$: C, 76.23; H, 6.96; N, 7.84 and found C, 76.18; H, 6.91; N, 7.78. **FT-IR (ATR), υ** cm^{-1}: 3341 (-O-H stretching), 2956, 2868 (C-H stretch), 2216 (C≡N stretch), 1552, 1500 (C=C stretch). **Mass (m/z):** Calculated: 535.68; Obtained (M-H): 534.20.

The detailed structural characterization details of molecules PO1, PO2, PO4, and PO5 and their intermediates, were outlined in our previous publication[1]. UV-Vis absorption spectra were recorded at a concentration of 10^{-5} M in chloroform ($CHCl_3$) solutions using a Labman UV-Vis spectrophotometer. The DNA binding affinities of the dyes were assessed using electronic spectroscopy in Tris-HCl buffer (10 mM, pH 7.45) with calf thymus DNA (CT-DNA). Computational studies were performed for isolated dyes in the gas phase using Biovia Turbomole 2022 software[2].

Identification of a protein target for docking analysis

The proteins CT-DNA (PDB ID: 1BNA, Resolution:1.90 Å, R-Value Observed: 0.178), (RCSB PDB: Homepage) and are the macromolecules chosen for docking studies [3]. The Biovia Discovery Studio visualizer software was employed to convert the macromolecules into pdb format, and docking experiments were performed using the 3D structure. The Autodock Vina 4.2 software is employed in molecular docking research to predict the interactions between compounds and target protein [4]. The evaluation of protein-ligand interactions will focus on binding affinity and bond length. Key elements of docking scores encompass the binding of amino acids and the formation of hydrogen bonds. The energy is assessed by evaluating the Root Mean Square Deviation (RMSD) of the ligand-protein interaction. [5]

Ligand preparation

Compounds were retrieved from PubChem compound database in form of Sdf file (https://pubchem.ncbi.nlm.nih.gov/), using of chemsketch/ACD (ChemSketch | ACD/Labs (acdlabs.com)) to convert the mol format[6]. The Biovia Discovery Studio visualizer tool was used the conversion of into pdb chemical format, and docking experiments were conducted utilizing 3D structure. The Autodock Vina software was utilized to set the internal levels of torsions, add mobility and polar hydrogens, and mix non-polar hydrogens with carbons. The dockable PDBQT format was further created from the ligand molecules [7].

Drug-likeness prediction of ligands:

The potential of compounds as pharmaceutical agents is assessed through the use of Molinspiration, which calculates molecular properties and bioactivity scores (Calculation of molecular properties and bioactivity score (molinspiration.com)). The Rule of 5, also known as Lipinski's Rule of Five, comprises a set of criteria employed in drug discovery to predict the oral bioavailability of drug candidates. Molecular Weight (MW): A prospective drug candidate should have a molecular weight (Da) of less than 500. Hydrogen Bond Donors: The number of hydrogen bond donors, which can be nitrogen or oxygen, should not exceed five. Hydrogen Bond Acceptors [8–10]: The total number of nitrogen or oxygen atoms acting as

hydrogen bond acceptors should not exceed ten. Log P (Partition Coefficient): The log P partition coefficient should be less than 5. Log P serves as an indicator of a compound's lipophilicity, which reflects its preference for lipid-rich environments. Lower Log P values are associated with enhanced oral bioavailability [11].

SwissADME properties of ligands

The SwissADME web server can be utilized to forecast the ADME properties and toxicity of compounds. This platform evaluates various atomic characteristics related to anti-cancer activity, human oral bioavailability, gastrointestinal absorption, blood-brain barrier permeability, and interactions with several cytochrome P450 enzymes, including CYP1A2, CYP2C19, CYP2C9, CYP2D6, and CYP3A4, as well as P-glycoprotein substrates and skin permeation (Log Kp). Following this analysis, molecular docking techniques are employed to experimentally assess the efficacy of these compounds. [12]

Table S1. The binding affinity of CT-DNA against compounds

Compound	Type of bond	Binding affinity (kcal/mol)	Amino acid	Bond length (Å)	Binding site of the Protein	Binding site of the Ligand
**PO1**	H -bond<br>H -bond<br>H -bond	-8.7	A: DG5<br>A: DA7<br>B: DA19	1.95<br>2.45<br>2.52	N9<br>Pyrimidine Ring (C1-C4)<br>N7	N7<br>Pyrimidine Ring<br>Pyrimidine Ring
**PO2**	H -bond<br>H -bond<br>H -bond<br>H –bond<br>H -bond<br>H -bond<br>H -bond<br>H-bond	-8.1	B: DA19<br>A: DG10<br>B: DG16<br>B: DG17<br>A: DG11<br>A: DC9<br>A: DC8<br>A: DA17	1.87<br>2.24<br>2.32<br>2.34<br>2.45<br>2.11<br>2.67<br>5.22	O1<br>Pyrimidine Ring (C1-C4)<br>Pyrimidine Ring<br>Pyrimidine Ring<br>Pyrimidine Ring (C1-C4)<br>N9<br>Pyrimidine Ring<br>Pyrimidine Ring	Pyrimidine Ring<br>N4<br>N9<br>Pyrimidine Ring<br>Pyrimidine Ring<br>N7<br>N9<br>Pyrimidine Ring
**PO4**	H -bond<br>H -bond<br>H –bond<br>H -bond	-8.0	A: DG5<br>A: DG4<br>B: DG22<br>B: DG23	2.29<br>2.40<br>2.58<br>2.66	Pyrimidine Ring<br>N2<br>Pyrimidine Ring<br>N4	N9<br>Pyrimidine Ring<br>Pyrimidine Ring<br>O2
**PO5**	H -bond<br>H -bond<br>H -bond<br>H -bond<br>H -bond	-10.7	A: DA7<br>B: DA19<br>A: DC8<br>A: DG4<br>B: DG22	2.05<br>2.15<br>2.49<br>2.76<br>3.24	Pyrimidine Ring (C1, C2,C4)<br>Pyrimidine Ring<br>Pyrimidine Ring<br>Pyrimidine Ring<br>N4	N4<br>N9<br>Pyrimidine Ring<br>Pyrimidine Ring<br>Pyrimidine Ring

DFT Simulation calculation

Cartesian coordinates of the PO1 dye

ATOM CARTESIAN COORDINATES

1 c	7.01827488428569	-6.10693000831467	0.34335887872592
2 n	5.89234820463369	-3.59827229694656	0.56094608380246
3 c	7.43416637132707	-1.45681685806451	0.56709450706574
4 c	6.32764551137446	0.91077502666798	1.04693517388257
5 c	7.74379725294375	3.08768663450953	1.08559563276735
6 c	10.35789803329542	2.96927108524041	0.66966217694812
7 c	11.82478306386075	5.33912092795974	0.75204436088789
8 o	14.31452505312957	5.00271385426517	0.33564822769883
9 o	10.92394798888076	7.41911311469009	1.15245080056419
10 c	11.47968373264834	0.63682197295464	0.17707801399150
11 c	10.03235027517917	-1.55080372014941	0.11103815581337
12 o	3.78215914066234	1.09788653790222	1.58097449489821
13 c	2.25325961848010	-0.87727625852084	0.80811406171202
14 c	3.31414977928402	-3.27748657589120	0.32375758804130
15 c	1.66365927924305	-5.22674346963519	-0.37756484773864
16 c	-0.90059515910154	-4.78380242328277	-0.55067247520100
17 c	-1.97305859846290	-2.39689262621279	-0.01893469214278
18 c	-4.65765747665962	-2.24983618575938	-0.32590021655637
19 c	-6.56847836425527	-0.53203895261703	0.06359710036477
20 c	-9.08130954636993	-1.40411873285942	-0.80088156089847
21 n	-11.07346092713773	0.29095841800883	-0.56116689622055
22 c	-13.60512789116089	-0.43440785054083	-1.44142102303378
23 c	-10.86263170828902	2.68009434166108	0.48435832039357
24 n	-8.51678763526257	3.41795386100150	1.37085446325642
25 c	-8.40301379241047	5.93439300128868	2.54004288143458
26 c	-6.33325589637144	1.93616642051163	1.27829012139100
27 o	-4.37711502717750	2.74357188614714	2.22311524152096

28 o	-12.69212504094934	4.07914206963647	0.63494103376222
29 o	-9.45418691799695	-3.50086628198736	-1.71844222895712
30 c	-0.28773265298693	-0.42768058252430	0.64204623687735
31 h	5.74548061054422	-7.49185919185488	1.16422271096010
32 h	8.76959648433102	-6.15159024633431	1.41344041837024
33 h	7.40244214240508	-6.60596907436278	-1.62364178262928
34 h	6.83027710824919	4.87454300017490	1.46998647621842
35 h	15.14356958981163	6.63932216645103	0.41762686483075
36 h	13.48766038911621	0.51368207482467	-0.16885024160598
37 h	10.93949584201409	-3.32794130298025	-0.31025525217443
38 h	2.38859370225406	-7.07884446438537	-0.82534734085387
39 h	-2.12496271141133	-6.32007649935527	-1.12092407049972
40 h	-5.40644252609028	-3.98690613194993	-1.11722128581071
41 h	-14.25395707124211	0.90261226878850	-2.86256811607614
42 h	-14.92050877927907	-0.43702469908739	0.14027243601655
43 h	-13.47324429268871	-2.31123627487877	-2.24652034516986
44 h	-6.46010681493145	6.29000801541157	3.07505044115972
45 h	-9.03776472165517	7.35122732894578	1.19260963335597
46 h	-9.61075993053931	5.98957607067734	4.20476827034202
47 h	-0.99548057552312	1.42878063077541	1.07714501706879

Cartesian coordinates of the PO2 dye

ATOM	CARTESIAN COORDINATES		
1 c	8.07077936412604	-1.50367534312676	-5.05726170999585
2 n	7.43007125179061	-0.71502107970109	-2.49173186172005
3 c	9.26668463053795	0.40099996405836	-0.95924232520371
4 c	8.68760242857903	0.87927424172941	1.58688755565294
5 c	10.41844732719422	1.96320214889652	3.19725690029614
6 c	12.83679475600785	2.59179975640389	2.31182174803311
7 c	14.78925550301579	3.75980817746094	3.94404464078974
8 o	14.43366249480118	3.70613955611259	6.47103914867841
9 o	16.68730311525699	4.76495313778977	3.13508765205034
10 c	13.41856351934594	2.15856941240335	-0.22061193767595
11 c	11.66222312104702	1.07846137597459	-1.83867596828990

12	o	6.36813456708185	0.21303142883560	2.56812301107986
13	c	4.41829437955231	-0.13239341715272	0.86142585148657
14	c	4.94771722127844	-0.66066333706581	-1.69775812525003
15	c	2.88885323088229	-1.11033565969892	-3.30705640469997
16	c	0.43521104137203	-1.05633919300481	-2.42010137940006
17	c	-0.10307831235570	-0.52547999868844	0.13532777715122
18	c	-2.51650988767197	-0.39316636223835	1.34293564954353
19	c	-5.03801566325600	-0.58576922088702	0.71554321207769
20	c	-6.77622504637002	-0.27008408053724	2.86153157881952
21	n	-9.37208995662283	-0.43349213745396	2.36418656043080
22	c	-11.04633374417758	-0.17709665418763	4.59793397911752
23	c	-11.68752783088268	2.56814592091800	5.16391324242663
24	c	-10.39585554516352	-0.67870840679971	-0.01318256991037
25	n	-8.74595120123940	-0.92422209798028	-2.00325002294351
26	c	-9.73469924456106	-1.24794235911117	-4.60563569589468
27	c	-10.16821955261201	-4.01336813972304	-5.26635732781092
28	c	-6.09407136832528	-1.03502286082881	-1.77543673763936
29	o	-4.84481420335871	-1.46609294801647	-3.67562700200273
30	s	-13.55629015484535	-0.68535632524210	-0.46027516158169
31	o	-6.06860439772214	0.09954735024412	5.03593107043194
32	c	1.99485289435192	-0.04815816215975	1.74781493868015
33	h	9.97722894198788	-2.26497582683158	-5.06782568535581
34	h	7.94934043111948	0.06701505862034	-6.39222241610059
35	h	6.79398233297894	-2.99970276543687	-5.64293624367092
36	h	9.81692016987940	2.30681234347181	5.12241490256619
37	h	12.97141076579657	2.71528585589036	6.95059720319549
38	h	15.26600730613014	2.66588829555956	-0.92835005250587
39	h	12.16299691948217	0.78327139656538	-3.79324847272729
40	h	3.20876233662628	-1.48762188746798	-5.28588927462046
41	h	-1.10027026963993	-1.41688479401890	-3.70156729165682
42	h	-2.32040895568749	-0.01024787145795	3.34775291675392
43	h	-10.04245367114047	-1.03002536463645	6.16945607755780
44	h	-12.73664269778611	-1.26701770038944	4.20813313601885
45	h	-9.98210419907278	3.65876999889368	5.55115765880038

46 h	-12.68979503177795	3.42865025152603	3.58233727048851
47 h	-12.90380841863369	2.65575076261758	6.82813942915199
48 h	-11.47780957462945	-0.17890207710518	-4.72062467291831
49 h	-8.34634194315100	-0.40893478546899	-5.85987590376902
50 h	-10.88758860455474	-4.14916764001956	-7.19543133414269
51 h	-11.55402788514579	-4.86243470470143	-3.99885856476239
52 h	-8.41036745801517	-5.08225506793712	-5.15304936928352
53 h	1.69880476817582	0.35518183510313	3.73129040025348

Cartesian coordinates of the PO4 dye

ATOM	CARTESIAN COORDINATES		
1 c	7.11481394459165	3.92104447027951	3.80520025006467
2 n	5.82785043378718	2.01082248147686	2.29228872487568
3 c	7.21715402158037	0.32262058951371	0.83083415371401
4 c	5.93773352591736	-1.65501149962595	-0.40404561315373
5 c	7.19762395480950	-3.39014314060070	-1.86423741919741
6 c	9.83470522534376	-3.25629231027650	-2.10915306719885
7 c	11.09510476850846	-5.17920835040626	-3.70102432433789
8 o	13.64057788763583	-5.34571158765334	-3.59846394761097
9 o	9.96842322450734	-6.61300046915690	-5.09673249157255
10 c	11.12347445857497	-1.29162649321699	-0.91654286017889
11 c	9.83432111460609	0.48443632473856	0.52195512846830
12 o	3.36229779472662	-1.95138785940096	-0.10467773391104
13 c	2.01044947965979	0.14872549651225	0.65410845493016
14 c	3.23585721061729	2.15415150598181	1.90599327911126
15 c	1.75323997916936	4.19157358708273	2.70389803733688
16 c	-0.83215061886182	4.19286825341462	2.29887769499062
17 c	-2.05428987667991	2.18747357823297	1.06241472311948
18 c	-4.74511277501704	2.35276626725859	0.73388399927270
19 c	-6.42200389956487	0.73832665928858	-0.32319991084538
20 s	-5.89124809865763	-2.25371265650317	-1.73324645373755
21 c	-9.13735041556969	-2.63249636677379	-2.43160030218302
22 n	-10.48101502702946	-0.59365141994083	-1.63364401236702
23 c	-13.24311478367813	-0.40934801287771	-1.88771923277971

24 c	-14.60158126277673	-1.56366300054298	0.37117928147026
25 c	-9.12689864841408	1.35057425867854	-0.42833881578980
26 o	-10.14928019651961	3.24920838910986	0.38329998100033
27 s	-10.24720589535110	-5.19203756752417	-3.87656396414667
28 c	-0.54473583322827	0.15598296200140	0.21860818775413
29 h	5.90685553907202	4.46447932966917	5.37404250139706
30 h	8.83441730172471	3.11563752454416	4.58573665882967
31 h	7.58696632383925	5.59655535735484	2.69208277106051
32 h	6.14693870415436	-4.88434604371280	-2.77909361421869
33 h	14.35726564366368	-4.24851095034838	-2.32058747541634
34 h	13.14704638667863	-1.05773324994732	-1.11135666366134
35 h	10.88054805811462	2.00894488524141	1.38158045574507
36 h	2.61479667172212	5.79016469653270	3.63256606913347
37 h	-1.93595148698267	5.78942301086083	2.94511425867782
38 h	-5.60239577783758	4.06756165098238	1.46568311646224
39 h	-13.67361685445733	1.59161589063686	-2.07181329470647
40 h	-13.75015663407796	-1.36570980067192	-3.63417785650185
41 h	-14.14755789810682	-3.56545900105907	0.55585211186104
42 h	-16.63910524635970	-1.38012591451311	0.11024160446872
43 h	-14.08032808760401	-0.59742566753605	2.11581357862575
44 h	-1.32336233622809	-1.45835580710259	-0.75709365553279

Cartesian coordinates of the PO5 dye

ATOM	CARTESIAN COORDINATES		
1 c	9.47875706377378	5.43805584306679	-0.07324546681229
2 n	8.65115304064161	2.83689060343425	-0.44376252667878
3 c	10.37141538715102	0.86152240251942	-0.24522725946450
4 c	9.57030345793113	-1.60164862199222	-0.85822964123705
5 c	11.18159474978125	-3.62974958487661	-0.72324604366294
6 c	13.70060669493604	-3.28278668322465	0.03459345647616
7 c	15.36648738144906	-5.52644670233968	0.08580466297396
8 o	17.64507518348239	-5.31017252179413	1.21956501260791
9 o	14.80395251066009	-7.55280578188491	-0.83751433531189
10 c	14.51750091138330	-0.85466889396468	0.64993020240718

11	c	12.87474244030819	1.18836213075158	0.53084112597330
12	o	7.14138776689963	-2.02359725795662	-1.71564193124055
13	c	5.34724313099125	-0.22709901273057	-1.08714916202630
14	c	6.08606357478188	2.24938854828249	-0.47954897975693
15	c	4.18025454614533	4.00601370892235	0.06052412690687
16	c	1.65698172239697	3.32424067543872	-0.04519652478282
17	c	0.92437293949618	0.85168048888066	-0.66509829352489
18	c	-1.67579226796070	0.02987519795748	-0.80225661541844
19	c	-3.76441110961747	1.46587600392526	-0.42867822695794
20	c	-6.33633911547862	0.58885952824134	-0.56468990840969
21	c	-8.35558977693228	2.58935125243239	-0.49794639691871
22	c	-10.94885541036834	1.64128024021314	0.39567466943368
23	c	-10.90137614887919	1.08791308440278	3.24446323562297
24	c	-12.95718465064093	3.64961170109019	-0.15649196614751
25	c	-11.58482988944012	-0.77505899361107	-1.08762243746468
26	c	-6.98893552091458	-1.91228312467974	-0.76243653507865
27	c	-9.52548696182052	-2.72271967176564	-0.96713186462505
28	c	-10.13339579549371	-5.27494389341617	-1.11593229731265
29	c	-12.66214370521014	-6.12286075302123	-1.33025165081425
30	n	-14.72093224434809	-6.83995872853135	-1.50750853468697
31	c	-8.25522390410634	-7.18503348397258	-1.08802911867491
32	n	-6.71909319449301	-8.74059600599588	-1.06096899077830
33	c	2.84787837616400	-0.92384495673694	-1.16030979314041
34	h	9.55746586678792	5.94707396946621	1.92801566508531
35	h	8.18625506432338	6.70195043737063	-1.04623935530205
36	h	11.33928307512444	5.67903509108698	-0.90900818266375
37	h	10.49884917987608	-5.48853992897637	-1.22581825031149
38	h	17.85966649055276	-3.69023585222587	2.04437742982689
39	h	16.44890371876797	-0.47830119648633	1.21183139339026
40	h	13.56032913022738	3.03877385560300	1.04493873919097
41	h	4.66976484585960	5.91616725620043	0.58347147218390
42	h	0.25031723628420	4.74491029702275	0.37814108255786
43	h	-1.91529456420757	-1.95137802728838	-1.27411221575141
44	h	-3.54343998421624	3.46222720141612	-0.02448668486018

45 h	-8.53239272003891	3.35785834321964	-2.41652363131643
46 h	-7.72304704232749	4.15850413408972	0.68642683909933
47 h	-9.47908997202119	-0.32280103765120	3.73420678160801
48 h	-12.73922753594051	0.38624774588491	3.87019852385300
49 h	-10.47945251606417	2.81065806811397	4.30004374407997
50 h	-14.82072248315150	3.00603259185993	0.45712474463051
51 h	-13.05086950411526	4.07063558204544	-2.17571443624408
52 h	-12.52554914049119	5.40161376515035	0.84774420910056
53 h	-11.85563570146008	-0.29093491879378	-3.08496455967064
54 h	-13.37115993116137	-1.56230498520609	-0.42570921041740
55 h	-5.52942724156748	-3.34435331459865	-0.73455266352683
56 h	2.37829254628713	-2.85548581436913	-1.64608942108124

References

(1) Keremane, K. S.; Planchat, A.; Pellegrin, Y.; Jacquemin, D.; Odobel, F.; Vasudeva Adhikari, A. Push-Pull Phenoxazine-Based Sensitizers for p-Type DSSCs: Effect of Acceptor Units on Photovoltaic Performance. ChemSusChem 2022, 15 (16), e202200520. https://doi.org/10.1002/CSSC.202200520.

(2) Furche, F.; Ahlrichs, R.; Hättig, C.; Klopper, W.; Sierka, M.; Weigend, F. Turbomole. Wiley Interdiscip Rev Comput Mol Sci 2014, 4 (2), 91–100. https://doi.org/10.1002/wcms.1162.

(3) Mohanty, M.; Mohanty, P. S. Molecular Docking in Organic, Inorganic, and Hybrid Systems: A Tutorial Review. Monatsh Chem 2023, 154 (7), 683–707. https://doi.org/10.1007/S00706-023-03076-1.

(4) Gouthami, K.; Veeraraghavan, V.; Nagaraja, P. In-Silico Characterization of Phytochemicals Identified from Vitex Negundo (L) Extract as Potential Therapy for Wnt-Signaling Proteins. Egyptian Journal of Medical Human Genetics 2022, 23 (1), 1–15. https://doi.org/10.1186/S43042-022-00219-7.

(5) Masagalli, J. N.; Mahadevan, K. M.; Siddalingmurthy, E.; Bagchi, P. Synthesis and Molecular Docking Studies of 3-Methyl-1,4-Diarylazetidin-2-Ones. Indian Journal of Chemistry (IJC) 2024, 63 (11), 1155–1163. https://doi.org/10.56042/IJC.V63I11.13328.

(6) M Mallikarjunaswamy, A. M.; Naik, P.; Bharath Kumar, M.; Gouthami, K.; Damodara Reddy, V.; Nair, V. A. Synthesis and Antimicrobial Evaluation of 2-Thioxoimidazolidinone Derivatives. Org. Commun 2024, 17, 178–192. https://doi.org/10.25135/acg.oc.174.21.3340.

(7) Kumari, D.; Vhanmane, V. R.; Gouthami, K.; Reddy, V. D.; Nagendra, G. 2-DPC Mediated Effective Synthesis of Peptide Conjugates, Their Antifungal and Antibacterial Properties. ChemistrySelect 2024, 9 (31), e202402123. https://doi.org/10.1002/SLCT.202402123.

(8) Babu, D. D.; Saranga Pani, A.; Joshi, S. D.; Naik, P.; Jayaprakash, G. K.; Al-Ghorbani, M.; Rodrigues, B.; Momidi, B. K. Computational and Experimental Insights into Pharmacological Potential: Synthesis, in Vitro Evaluation, and Molecular Docking Analysis

of Bioactive Urea and Thiourea Derivatives. Microb Pathog 2025, 200, 107209. https://doi.org/10.1016/J.MICPATH.2024.107209.

(9) Bharath kumar, M.; Hariprasad, V.; Joshi, S. D.; Jayaprakash, G. K.; Parashuram, L.; Pani, A. S.; Babu, D. D.; Naik, P. Bis(Azolyl)Pyridine-2,6-Dicarboxamide Derivatives: Synthesis, Bioassay Analysis and Molecular Docking Studies. ChemistrySelect 2023, 8 (12), e202204927. https://doi.org/10.1002/SLCT.202204927.

(10) Kumar, M. B.; Hariprasad, V.; Joshi, S. D.; Naik, P.; Jayaprakash, G. K.; Pani, A. S.; Babu, D. D. Exploring the Antimicrobial Potential of Pyrimidine Linked Hydrazinyl Azole Derivatives: Insights from Biological Assays and Molecular Docking Studies. ChemistrySelect 2023, 8 (44), e202301998. https://doi.org/10.1002/SLCT.202301998.

(11) Kenny, P. W. Hydrogen-Bond Donors in Drug Design. J Med Chem 2022, 65 (21), 14261–14275. https://doi.org/10.1021/ACS.JMEDCHEM.2C01147.

(12) Kim, K. H.; Rateb, M.; Hassan, H.; Zhao, T.; Li, C.; Wang, S.; Song, X. Green Tea (Camellia Sinensis): A Review of Its Phytochemistry, Pharmacology, and Toxicology. Molecules 2022, Vol. 27, Page 3909 2022, 27 (12), 3909. https://doi.org/10.3390/MOLECULES27123909.
